# LncRNA00978 contributes to growth and metastasis of hepatocellular carcinoma cells via mediating microRNA-125b-5p/SOX12 pathway

**DOI:** 10.1080/21655979.2022.2063648

**Published:** 2022-04-29

**Authors:** Zhiqing Cheng, Limei Gong, Qinghe Cai

**Affiliations:** a Department of Hepatopancreatobiliary Surgery, Affiliated Hospital of Putian University, Putian, Fujian, China; b Department of General Medicine, Affiliated Hospital of Putian University, Putian, Fujian, China

**Keywords:** Invasion, hepatocellular carcinoma, migration, LINC00978, microRNA-125b-5p, SOX12, cell proliferation

## Abstract

As a malignant tumor, HCC (hepatocellular carcinoma) is featured by a high recurrence rate with a poor prognosis. Increasing evidence supports an important role of lincRNAs in HCC. Here, the purpose of the study was to explore the function of LINC00978 (long non-coding RNA00978) in HCC and the underlying mechanisms. LINC00978 expression and its association with the progression of HCC were analyzed using HCC TCGA datasets. LINC00978 expression in tissues was measured using real-time PCR. Then, we knocked down LINC00978 in HCC cells to explore its effect on cellular invasion, proliferation, and migration. Finally, we investigated the potential molecular mechanism of LINC00978 by dual Luciferase reporter assay, FISH (fluorescence in situ hybridization) and RIP (RNA immunoprecipitation). LINC00978 expression was remarkably increased in HCC. A high level of LINC00978 was associated with poor prognosis of HCC. Additionally, LINC00978 silencing could repress the growth and metastasis of HCC cells. Mechanistically, it was revealed that LINC00978 could sponge microRNA-125b-5p and identified SOX12 (SRY-Box Transcription Factor 12) as a direct target gene of microRNA-125b-5p. More importantly, the suppressed effect of LINC00978 silencing on the metastasis and growth of HCC cells could be rescued by miR-125b-5p inhibition and overexpressed SOX12. LINC00978/microRNA-125b-5p/SOX12 axis promoted liver cancer migration, invasion, and proliferation, which could be used as a possible therapeutic target for the treatment of hepatocellular carcinoma.

## Graphical Abstract

**Figure:**
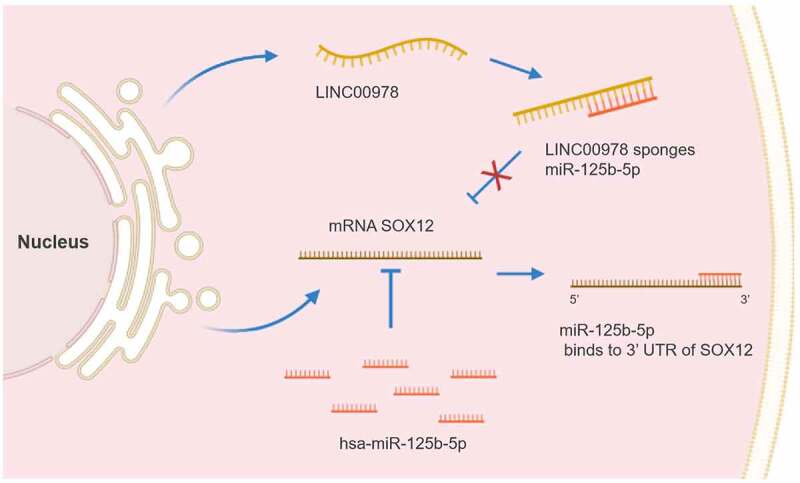

## Highlights

- LINC00978 is highly expressed in HCC;
- LINC00978 knockdown represses HCC cellular growth and cancer metastasis;
- LINC00978 works as a ceRNA to sponge microRNA-125b-5p in HCC;
- SOX12 gene is a potential target for miR-125b-5p;
- LINC00978 depletion represses HCC cellular growth and cancer metastasis via microRNA −125b-5p/SOX12 axis

## Introduction

HCC (hepatocellular carcinoma) accounts for 90% of primary liver malignancies [1,2]. It is currently believed that the occurrence of HCC is highly correlated with certain chemical carcinogens, such as viral hepatitis, aflatoxin, liver cirrhosis, and the factors of soil and water [3,4]. Clinically, HCC is characterized by liver pain, weight loss, fever, loss of appetite, and splenomegaly [5]. In terms of clinical treatment, liver transplantation, tumor ablation, transarterial treatment, and systemic treatment have been recently used to alleviate HCC. However, the high recurrence and metastasis rates of HCC are responsible for its poor prognosis. Thus, it is urgent to investigate the underlying mechanism of tumorigenesis, and more importantly, identification of the possible markers in predicting HCC recurrence is critical for attenuating disease development.

MicroRNAs and lncRNAs (long non-coding RNAs) belong to ncRNAs (non-coding RNAs). MicroRNAs and lncRNAs significantly contribute to the pathogenesis and progression of HCC [6,7]. Specifically, Zhang et al. [8] revealed that lncRNA-CCDC144NL-AS1 could sponge microRNA-940 and inhibit WD Repeat Domain 5 (WDR5) expression, thereby accelerating the progression of hepatocellular carcinoma. Wang et al. [9] suggested that long non-coding RNA X Inactive Specific Transcript (lncRNA XIST) promoted the development of hepatocellular cancer via mediating microRNA-192/Tripartite Motif Containing 25 (TRIM25) axis. Conversely, Chen B et al. found that LncRNA TP53TG1 negatively regulated cellular development and metastasis of hepatocellular cancer in a Peroxiredoxin 4 (PRDX4)/β-catenin pathway-dependent manner [10]. In addition, the function of LINC00978 (long non-coding RNA00978) [11] and microRNA-125b-5p (microRNA-125b-5p) [12] in HCC was, respectively, reported. Nevertheless, the specific mechanism of LINC00978 mediating microRNA-125b-5p and affecting the development of HCC remains elusive.

SOX12 (SRY-Box Transcription Factor 12), a member in the SOX (sex determining region Y-box) family, is characterized by a HMG (high mobility group) sequence with highly conserved properties [13]. According to the HMG sequences and other structural motifs, SOX family can be categorized into groups of A to H [14]. It has been proved that SOX gene (SOX11, SOX12) plays a role in the development of neuronal and EMT (epithelial–mesenchymal transition) [15,16]. Recently, accumulating studies have proved that SOX12 is related to the development of multiple malignancies, like myeloma [17], breast cancer [18] and glioma [19]. In addition, Wang et al. [20] suggested that microRNA-370 suppressed tumor growth and EMT in bladder cancer via inhibiting SOX12 transcription. Interestingly, SOX12 has been recently proved to participate in the metastasis of HCC [21]. However, whether and how LINC00978/microRNA-125b-5p mediates SOX12 and affects HCC development have not been elaborated.

Therefore, we measured the expression of LINC00978, microRNA-125b-5p and SOX12 in HCC, and investigated the correlation between the two. Moreover, the function of LINC00978/microRNA-125b-5p/SOX12 axis in liver cancer growth and metastasis was revealed, which can be used as a new marker and potential therapeutic target for HCC.

## Materials and methods

### Cell culture and transfection

Hep3B, SNU423, and SNU449, three human liver cancer cell lines were purchased from ATCC (American Type Culture Collection, Manassas, America). ATCC-formulated medium supplemented with FBS (fetal bovine serum; 10%) (Gibco, Rockville, America) was used for cell culture. Liver Cancer Institute (Fudan University, Shanghai, China) provided MHCC-97 H cell line. Dulbecco’s modification of Eagle’s medium (provided by Thermo Fisher Scientific, Waltham, America) was used to incubate MHCC-97 H cell line. All cell lines were incubated at 37°C in a 5% CO2 incubator. GeneChem Corporation (Shanghai, China) designed and synthesized sh-LINC00978-1, sh-LINC00978-2, microRNA-125b-5p-mimics, microRNA-125b-5p-inhibitors, and pcDNA-SOX12 (Table 1). The Lipofectamine 3000 reagent was used (Vision 2000, 11,668–019, Invitrogen, Carlsbad, CA, USA) for cell transfection. The SNU423 and SNU449 cells were seeded in the six-well plate and incubated to reach a 60%~70% confluence. Then, 2–5 μg of plasmid were transfected. For miR-125b-5p inhibitors or mimic transfections, 50 nM final concentration inhibitors/mimics were transfected. Three days after transfection, the cells were submitted for RNA extraction, and RT-qPCR was used to determine the interference efficiency.

**Table 1.** Sequences of shRNA

sh-LINC00978-1	5’-3’	CACCGCCCAGATTTAAGGGCTATTTCAAGAGAATAGCCCTTAAATCTGGGCCTTTTTTG
sh-LINC00978-2	5’-3’	CACCGCCCAGATTTAAGGGCTATTTCAAGAGAATAGCCCTTAAATCTGGGCCTTTTTTG

### FISH (fluorescence in situ hybridization)

To measure LINC00978, FITC (fluorescein isothiocyanate)-UTP (provided by Roche, Basel, Switzerland) was used to label the fragment of LINC00978 by using mMESSAGE T7 Ultra In Vitro Transcription kit (purchased from Life Technologies, Gaithersburg, MD, USA) as per the instructions. In other words, the probes were hybridized with slides overnight and washed with saline-sodium citrate. A fluorescence microscope (Olympus, Tokyo, Japan) was employed to acquire the images.

### Luciferase reporter assay

Bioinformatics ENCORI and TargetScan database were, respectively, used to predict the binding site of microRNA-125b-5p and LINC00978, and also microRNA-125b-5p and SOX12. To detect the luciferase activity, mimics for NC or microRNA-125b-5p were co-transfected with LINC00978-MUT or LINC00978-WT, and SOX12-WT or SOX12-MUT into SNU423 and SNU449 cells as per the protocols. Dual-Luciferase Reporter Assay System (purchased from Promega, Madison, WI, USA) was used to detect luciferase activity.

### RIP (RNA immunoprecipitation)

RIP was employed to analyze the correlation of microRNA −125b-5p and LINC00978, and also the association of SOX12 and microRNA −125b-5p. Briefly, Anti-AGO2 (ab186733, 1:50, Abcam, Cambridge, America) and Magna RIP RNA-binding protein immunoprecipitation kit (provided by Millipore, Billerica, America) were utilized. RNA bound complexes were then measured by RT-PCR; meanwhile, Anti-IgG was adopted as isotype control.

### CCK-8 (Cell counting kit-8)

10% CCK-8 solution (provided by Sigma, St. Louis, America) was added into the SNU423 and SNU449 cells in 96-well plates. The reagent in the cell counting kit (Beyotime Biotechnology, Shanghai, China) was utilized for the measurement of absorbance at 450 nm after the cells were incubated for different time points (96, 72 48, 24 h).

### Colony formation assay

For the analysis of cell proliferation rate, SNU423 and SNU449 cells (1 × 10^3^ cells/well) were separately cultured in 6-well plates for 14 days. Paraformaldehyde (4%) was then utilized to fix the cells, followed by crystal violet (1%) staining. The visible colonies were counted. A microscope was employed to record the images.

### Transwell assay

For the assessment of cell migratory and invasive ability, into the upper chambers, we inoculated the SNU423 and SNU449 cells. Into the lower chambers, we added DMEM added with FBS (10%). After incubation for 24 h, paraformaldehyde (4%) was used to fix the cells after removing the cells, followed by crystal violet (0.1%) staining. The migratory and invasive cells were calculated. The microscope (Zeiss, Oberkochen, Germany) was employed to acquire the images.

### Wound healing

SNU423 and SNU449 cells were maintained in 12-well plates for 1 day, followed by scratching, which were then cultured for another 2 days. The photograph was obtained, and the migration rate was measured under a microscope.

### WB (Western blotting)

The total protein of SOX12 from SNU423 and SNU449 cells was extracted by RIPA (radioimmunoprecipitation assay) buffer (Beyotime, Shanghai, China). The concentration of total protein was measured by using the BCA (bicinchoninic acid) protein Assay Kit (CWBIO). SDS-PAGE (sodium dodecyl sulfate-polyacrylamide gel) was prepared and used for electrophoresis to separate the protein. The protein was then transferred onto PVDF (polyvinylidene fluoride) membranes (Millipore, Billerica, MA, USA) by using the Bio-Rad Mini PROTEAN 3 system (Bio-Rad, Hercules, CA, USA). The membranes were incubated with primary antibodies overnight after blocked for 1 h in PBS (phosphate buffered saline) covering milk (5%). Afterward, the anti-rabbit secondary antibodies conjugated with horseradish peroxidase were used for incubation with the membranes. The Amersham ECL Western Blotting Detection Kit was applied for the visualization of blot bands. GAPDH (ab8245, 1:1,000, Abcam, Cambridge, America) was adopted as an internal reference. The primary antibodies SOX12 (PA5-103,280, 1:1000) were provided by Thermo Fisher Scientific (located at Waltham, America).

### RT-PCR (real-time PCR)

TRIzol reagent (provided by TaKaRa, Tokyo, Japan) was applied to extract total cellular RNA. PrimeScript RT Reagent Kit (provided by TaKaRa, Tokyo, Japan) was used for RNA reverse-transcription. LINC00978, microRNA-125b-5p and SOX12 levels were measured using RT-PCR. GAPDH and U6 were, respectively, adopted as the normalization of relative gene expression. All the sequences of the primers are listed in Table 2.

**Table 2.** Sequences of PCR primers used in this study

Gene		Primer sequences
LINC00978	Forward(5’-3’)	AGGCCCCAGGGAATCTTTCA
	Reverse(5’-3’)	GCCTCTCCCTGAATAACTGGG
miR-125b-5p	Forward(5’-3’)	CAGTCCCTGAGACCCTAAC
	Reverse(5’-3’)	GTCCAGTTTTTTTTTTTTTTTCACAAG
GAPDH	Forward(5’-3’)	GGAGCGAGATCCCTCCAAAAT
	Reverse(5’-3’)	GGCTGTTGTCATACTTCTCATGG
SOX12	Forward(5’-3’)	AAGAGGCCGATGAACGCATT
	Reverse(5’-3’)	TAGTCCGGGTAATCCGCCAT

### Statistical analysis

Our results were described as average value ± standard deviation. Kaplan–Meier curves were plotted to evaluate the overall survival. The difference between groups was assessed by ANOVA (one-way analysis of variance) and Student’s t-test. P < 0.05 indicated significant difference.

## Results

### LINC00978 is highly expressed in HCC

**Figure 1.**
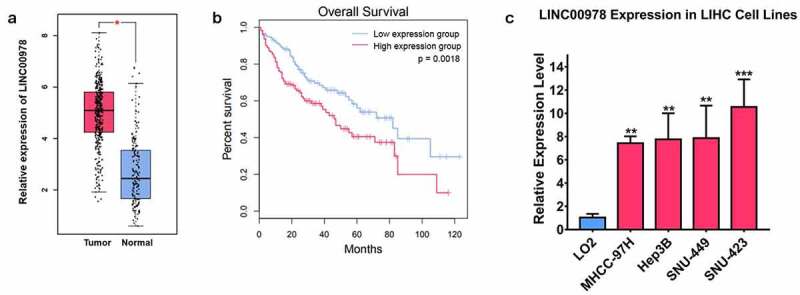
LINC00978 was upregulated in HCC. (a) TCGA database was used to evaluate the levels of LINC00978 in normal and hepatocellular carcinoma tissues. (b) The overall survival analysis of HCC patients based on LINC00978 expression. (c) LINC00978 levels in Hep3B, SNU423, SNU449 and MHCC-97 H, and human normal liver cells LO2 determined by qRT-PCR assay. *** P < 0.001, ** P < 0.01, * P < 0.05.

To investigate the relationship between HCC and LINC00978, the expression of LINC00978 was evaluated using TCGA database. In HCC and normal tissues, we found that LINC00978 was differently expressed (Figure 1(a)). Moreover, the overall survival analysis presented that the expression level of LINC00978 in HCC patients was related to worse overall survival (P < 0.01, Figure 1(b)). Moreover, we detected LINC00978 levels in HCC cell lines including SNU423, Hep3B, SNU449, and MHCC-97 H, and human normal liver cells LO2. The results showed LINC00978 levels were remarkably upregulated in HCC cell lines compared to LO2 cells (Figure 1(c)). In summary, the results above indicated that the expression level of LINC00978 was high in HCC. Upregulated LINC00978 was associated with a worse prognosis of HCC, demonstrating that LINC00978 might participate in the development of HCC.

### LINC00978 knockdown represses HCC growth and metastasis

**Figure 2.**
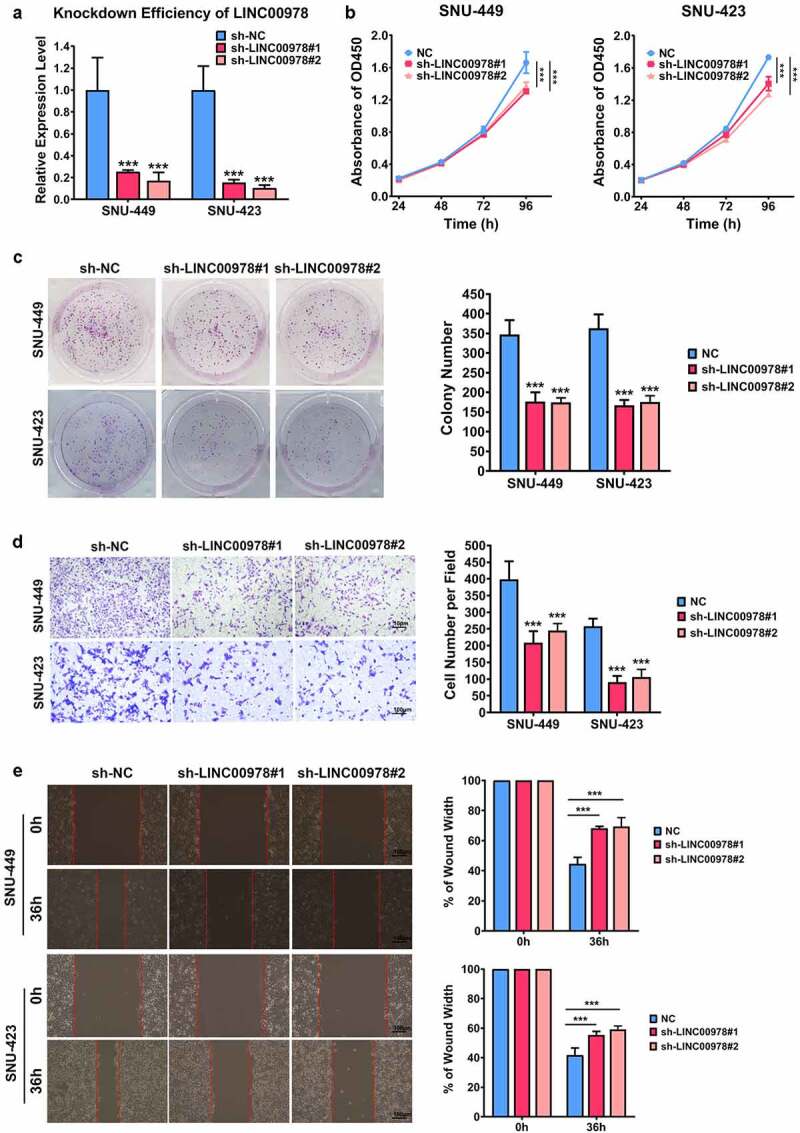
LINC00978 depletion represses HCC cellular growth and cancer metastasis. To investigate the function, SNU423 and SNU449 cell lines were transiently transfected with shRNA plasmids, and qRT-PCR was adopted to examine the transfected efficacy (a). Proliferation of SNU423 and SNU449 cells was assessed by CCK-8 (b) and colony formation assays (c). Transwell assay (d) and wound healing assay (e) were carried out to measure migration and invasion. ***P < 0.001.

To estimate the function of LINC00978 in hepatocellular carcinoma cells, we generated stable LINC00978-silenced SNU423 and SNU449 cells (Figure 2(a)). Colony formation assay (Figure 2(c)) and CCK-8 (Figure 2(b)) results revealed the inhibitory effect of LINC00978 silencing on HCC cell proliferation. Similarly, LINC00978 depletion could efficiently suppress invasion and migration capabilities of SNU423 and SNU449 cells (Figure 2(d, e)). Overall, our results suggested that LINC00978 knockdown markedly repressed invasion, proliferation, and migration of HCC cells.

### LINC00978 functions as a ceRNA and sponges microRNA-125b-5p in HCC

**Figure 3.**
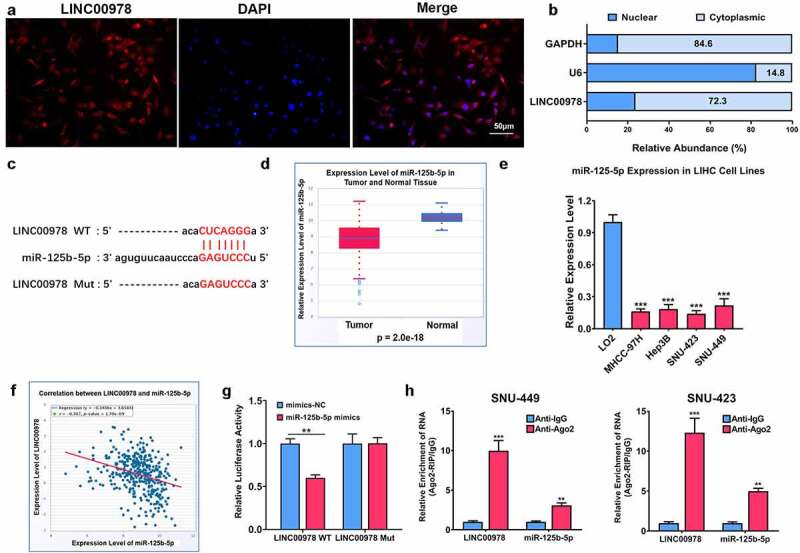
LINC00978 worked as a sponger for miR-125b-5p in HCC. (a) LINC00978 localization in hepatocellular carcinoma cells analyzed by FISH; (b) the relative LINC00978 level in the cytoplasmic and nuclear fractions of HCC cells revealed by qRT-PCR assay; (c) prediction of the binding site of LINC00978 and microRNA-125b-5p by ENCORI database; (d) levels of microRNA-125b-5p in normal and hepatocellular carcinoma tissues revealed by TCGA database; (e) microRNA-125b-5p levels in Hep3B, SNU423, SNU449 and MHCC-97 H, and human normal liver cells LO2 determined by qRT-PCR assay; (f) the correlation of LINC00978 and microRNA-125b-5p; the binding of LINC00978 and microRNA-125b-5p revealed by luciferase activity assay (g) and RIP assay (h). **P < 0.01, ***P < 0.001.

To reveal the possible mechanisms involved in mediating cellular biological function of HCC by LINC00978, we first determined LINC00978 localization at hepatocellular carcinoma cells using FISH. It was observed that LINC00978 was abundant in the cytoplasm (Figure 3(a)). Additionally, q-PCR assay presented that the relative expression of LINC00978 in the cytoplasmic fractions of HCC cells was up to 72.3% (Figure 3(b)), implying cytoplasmic LINC00978 might function as a ceRNA via competitively binding target microRNAs.

Subsequently, ENCORI database revealed a binding site of microRNA-125b-5p and LINC00978 (Figure 3(c)). Additionally, microRNA-125b-5p was differently expressed in normal and hepatocellular carcinoma tissues revealed by the TCGA database (Figure 3(d)). In hepatocellular carcinoma cell lines, microRNA-125b-5p levels were remarkably reduced in comparison with LO2 cells (Figure 3(e)). To further reveal the association of LINC00978 and microRNA-125b-5p, the correlation analysis was conducted, and it was found that there was a negative correlation between the two (Figure 3(f)). Furthermore, microRNA-125b-5p mimics efficiently restricted the activity of luciferase of a wild-type reporter gene rather than the gene containing mutant LINC00978 3’-UTR (Figure 3(g)). RIP assay further presented microRNA-125b-5p and LINC00978 were considerably abundant in the anti-AGO2 microribonucleoprotein complexes in SNU423 and SNU449 cells, in comparison with the anti-IgG group (Figure 3(h)). These results clarified that downregulated microRNA-125b-5p in HCC could sponge LINC00978.

### SOX12 is a target gene for microRNA-125b-5p

**Figure 4.**
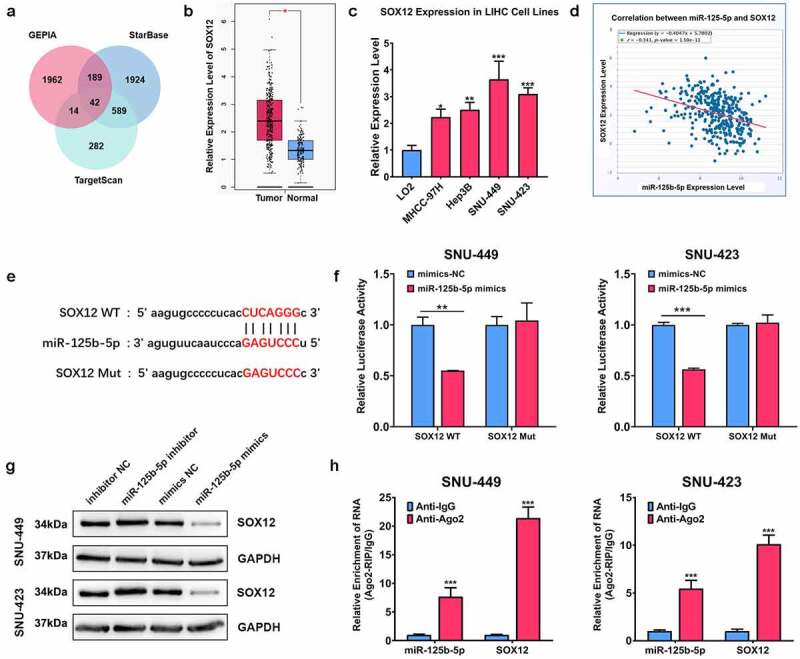
MicroRNA-125b-5p mediated SOX12 expression in HCC negatively. (a) To obtain the potential target for microRNA-125b-5p in HCC, GEPIA, StarBase and TargetScan databases were analyzed; (b) levels of SOX12 in normal tissues and HCC revealed by TCGA database; (c) SOX12 levels in Hep3B, SNU423, SNU449 and MHCC-97 H, and human normal liver cells LO2 revealed by qRT-PCR assay; (d) The correlation of SOX12 and microRNA-125b-5p; (e) prediction of the binding site of microRNA-125b-5p and SOX12 based on StarBase database; (f) the luciferase activity of SOX12 revealed by Luciferase reporter assay; (g) SOX12 levels in hepatocellular carcinoma cells after overexpressing/inhibiting microRNA-125b-5p revealed by Western blot assay; (h) the binding of LINC00978 and microRNA-125b-5p revealed by RIP assay. ***P < 0.001, **P < 0.01 *P < 0.05.

Our research next explored the potential target gene for microRNA-125b-5p in HCC cells. The intersection genes were predicted using GEPIA, StarBase, and TargetScan databases (Figure 4(a)), and it was found that the intersection gene SOX12 was highly expressed in HCC tissues (Figure 4(b)). Consistently, our results validated that the expression of SOX12 was increased in HCC cells (Figure 4(c)). Next, it was observed that there was a negative association between microRNA-125b-5p and SOX12 expression levels (Figure 4(d)), and there was a binding site between the two (Figure 4(e)). In addition, luciferase activity in the cells transfected with SOX12-WT plasmids was obviously reduced by microRNA-125b-5p mimics (P < 0.01). No change was observed in the cells after the transfection with SOX12-MUT plasmids (Figure 4(f)). Furthermore, downregulated microRNA-125b-5p efficiently increased SOX12 levels in SNU423 and SNU449 cells. Upregulated microRNA-125b-5p exerted a suppressed effect (Figure 4(g)). RIP assay clarified that microRNA-125b-5p and SOX12 were remarkably abundant in anti-AGO2 complexes in SNU423 and SNU449 cells, in comparison with the anti-IgG group (Figure 4(h)). In summary, our findings implied that microRNA-125b-5p suppressed SOX12 expression in HCC.

### LINC00978 depletion represses the growth of HCC cells and cancer metastasis through mediating the microRNA-125b-5p/SOX12 axis

**Figure 5.**
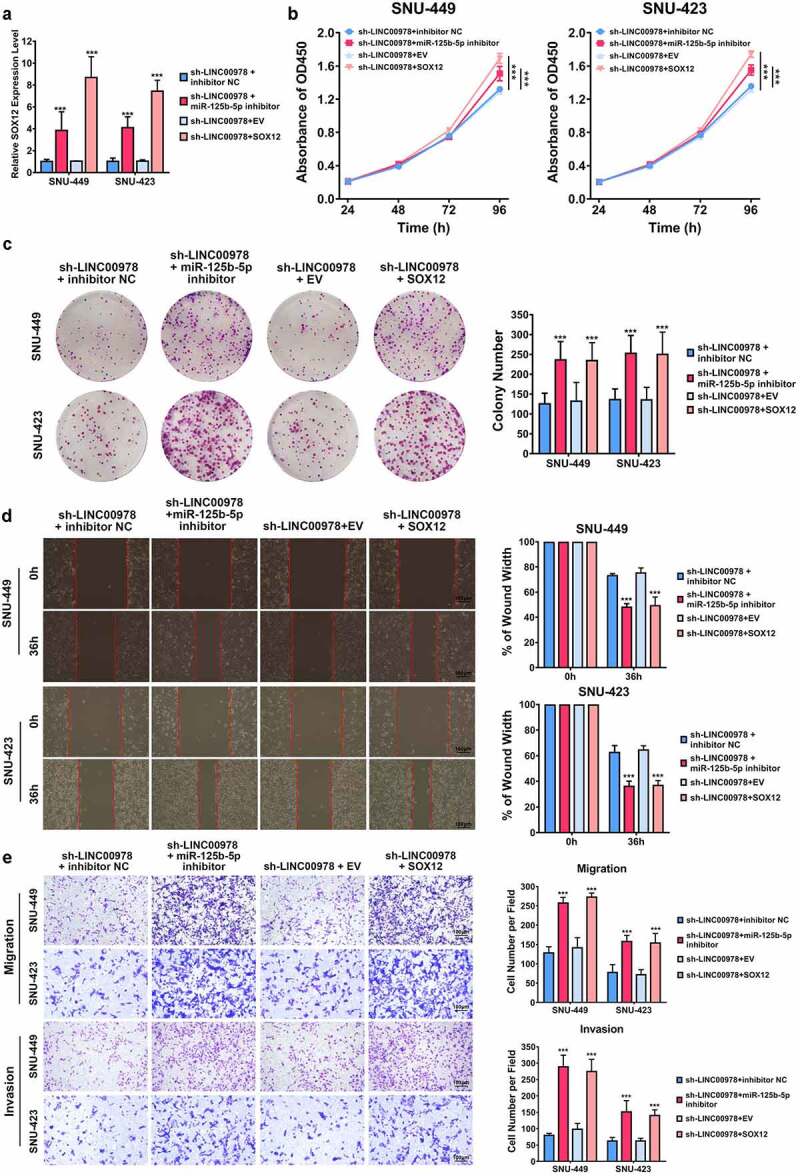
LINC00978 depletion repressed HCC cellular growth and cancer metastasis via microRNA-125b-5p/SOX12 pathway. (a) Co-transfection of LINC00978 and microRNA-125b-5p inhibitor or SOX12 overexpression plasmids in SNU449 and SNU423 cells, the SOX12 mRNA expression was determined by qRT-PCR. (b-d) Proliferation of SNU423 and SNU449 cells was assessed by CCK-8 (b) and colony formation assays (c). The migration and invasion were detected by wound healing (d) and transwell (e) assays. ***P < 0.001.

To validate if LINC00978 regulated HCC progression in a microRNA-125b-5p/SOX12 axis-dependent manner, we co-transfected LINC00978 and microRNA-125b-5p inhibitor or SOX12 overexpression plasmids in SNU449 and SNU423 cells, the SOX12 mRNA expression was determined by qRT-PCR (Figure 5(a)), which were then co-transfected with LINC00978 and microRNA-125b-5p depletion plasmids, as well as LINC00978 depletion and SOX12 overexpression plasmids. CCK-8 (Figure 5(b)) and colony formation (Figure 5(c)) assays indicated that downregulated LINC00978 attenuated the proliferation rate of SNU449 and SNU423 cells, and this effect was reversed by overexpressing SOX12 and inhibiting microRNA-125b-5p. Similarly, the suppressive role of LINC00978 silencing in HCC migratory and invasive capacities was retained by microRNA-125b-5p inhibition and SOX12 overexpression (Figure 5(d, e)). To sum up, these findings presented that the depleted LINC00978 repressed migration, proliferation, and invasion of HCC cells through mediating microRNA-125b-5p/SOX12 axis.

## Discussion

Our findings indicated that LINC00978 was upregulated in HCC, which was associated with a worse prognosis of HCC. Moreover, LINC00978 depletion repressed cellular growth and metastasis of HCC cells, and LINC00978 sponged microRNA-125b-5p, which could target SOX12 gene. Thus, LINC00978/ microRNA-125b-5p/SOX12 axis was related to HCC progression.

Aberrant oncogenes’ expression in HCC is a key feature in cancer initiation and development. Recently, accumulating biomarkers are identified for early detection and survival prediction in HCC [22–24]. Previous studies have suggested that LINC00978 is an oncogenic LncRNA in diverse cancers, like breast cancer [25], melanoma [26] and bladder cancer [27]. Our data presented that LINC00978 was upregulated in HCC. Moreover, it was also revealed that upregulated LINC00978 was closely associated with the worse prognosis of HCC, which was in accordance with previous studies. For example, Deng LL and other scholars suggested LINC00978 was upregulated in breast carcinoma cells, the increased expression of which predicted a poor prognosis [28]. In addition, accumulating studies have validated that LINC00978 significantly regulates cellular proliferation, apoptosis, metastasis, and inflammation. Specifically, Zhang et al. suggested that depleted LINC00978 efficiently repressed the proliferation of HCC cells, and meanwhile promoted the apoptosis and the arrest of cell cycle. Bu et al. [29] reported that downregulating LINC00978 could suppress cellular proliferation and tumor progression in gastric cancer. Consistent with previous reports, we demonstrated that shRNA-mediated LINC00978 knockdown could repress the growth and metastasis of HCC cells. With the development of multi-omics, especially the application of single-cell multi-omics and spatial transcriptomics in cancer research [30–33], we look forward to using these technologies for further investigation of LINC00978 in HCC development.

Apart from mediating DNA, RNA, or proteins, LncRNAs also served as a ceRNA of miRNA [27]. For instance, LINC00978 could facilitate the progression of breast carcinoma via working as a sponger for microRNA-4288. Li et al. [34] revealed the regulatory mechanism that LINC00978 facilitated cell development and cancer metastasis through sponging microRNA-6754-5p in non-small cell lung cancer. In addition, Ma et al. [26] proved that LINC00978 significantly enhanced melanoma progression via recruiting microRNA-802 from FLOT2. Similarly, we implied that microRNA-125b-5p levels were reduced in hepatocellular carcinoma, and involved in regulating the function of HCC cells by LINC00978. It was also found that LINC00978 could reduce microRNA-125b-5p levels via acting as its sponger.

As referred above, SOX12 is a member of the SOX family. Recent evidence proved that SOX12 played a part in the progression of multiple cancers. PRR34-AS1 promotes HCC proliferation and metastasis through targeting miR-296-5p/E2F2/SOX12 [35]. SOX12 transcriptionally targets matrix metallopeptidase 7 (MMP7) and insulin-like growth factor 1 (IGF1) to facilitate gastric cancer metastasis [36]. SOX12 enhances the Tumorigenic Properties and Chemoresistance in Cervical Cancer by targeting lncRNA SNHG15/miR-4735-3p/HIF1a Pathway [37]. In our study, SOX12 was overexpressed in cells and HCC tissues, which was mediated by microRNA-125b-5p in a negative way in HCC cells. In addition, it was found that the repressed effect by LINC00978 depletion on cellular growth and cancer metastasis in HCC was rescued by inhibiting microRNA −125b-5p and overexpressing SOX12.

## Conclusions

In conclusion, this research presented that LINC00978 overexpression was related to worse prognosis of HCC. Furthermore, depleted LINC00978 inhibited cellular invasion, proliferation, and migration in HCC. LINC00978 sponged microRNA-125b-5p in HCC cells, and SOX12 gene was a target for microRNA-125b-5p. What is more, LINC00978 knockdown could repress HCC cellular growth and cancer metastasis through mediating microRNA −125b-5p/SOX12 axis. Thus, LINC00978/microRNA −125b-5p/SOX12 axis may be a new target for the management of hepatocellular carcinoma.

## Abbreviations

CCK-8, cell counting kit-8; HCC, hepatocellular carcinoma; BCA, bicinchoninic acid; HMG, high mobility group; ATCC, American Type Culture Collection; LINC00978, long non-coding RNA00978; PBS, phosphate-buffered saline; RIP, RNA immunoprecipitation; PVDF, polyvinylidene fluoride; SOX12, SRY-Box Transcription Factor 12; SDS-PAGE, sodium dodecyl sulfate-polyacrylamide gel electrophoresis; ncRNAs, Non-coding RNAs; lncRNAs, long non-coding RNAs; FISH, fluorescence in situ hybridization; SOX, sex determining region Y-box; EMT, epithelial–mesenchymal transition; FBS, fetal bovine serum; FITC, fluorescein isothiocyanate; WB, Western blotting; RIPA, radioimmunoprecipitation assay; ANOVA, analysis of variance.
